# Relating Habitat Suitability and Survival Rates in a Phylogenetic Framework

**DOI:** 10.1002/ece3.71030

**Published:** 2025-03-02

**Authors:** Eun Hye Kim, James D. Hitchmough, Ross W. Cameron, Volker Bahn, Franziska Schrodt

**Affiliations:** ^1^ School of Geography University of Nottingham Nottingham UK; ^2^ School of Architecture and Landscape University of Sheffield Sheffield UK; ^3^ Department of Biological Sciences Wright State University Dayton Ohio USA

**Keywords:** climate tolerance, extrapolation, habitat suitability assessment, model transferability, niche similarity, phylogenetic signal, SDM

## Abstract

Species distribution models rely on species' observed geographic distributions, which reflect only subset of the true ecological niche. This inevitably leads to discrepancies between the predictions of habitat suitability (HS) and the actual ecological performance in novel environments beyond the trained range. We examined this limitation by comparing modelled HS with empirical survival rates (SRs) of three *Acer* species, *A. davidii, A*. *palmatum* , and *A. pictum*, cultivated in the UK botanic gardens. We hypothesise that ex‐situ species with greater niche overlap with native UK/European species will show higher HS, which also correspond to species' SR relative to that of local species. This HS‐SR alignment will then indicate the alignment of species' geographic range and ecological range. We first quantified niche similarity between these East Asian species and UK/Europe native *Acer* species at both regional and continental scales. MaxEnt models were calibrated using native occurrences with various combinations of environmental variables and model configurations, then projected onto UK regions. Species' SRs were standardised against those of native species using long‐term inventory data. Our results show that niche overlap with native species generally corresponded to predicted HS, while observed SR patterns revealed an inverse relationship. *A. davidii*, showing high niche overlap and high HS, exhibited the lowest SR. Contrarily, *A. pictum* , despite showing low niche overlap and predicting most regions unsuitable, demonstrated the highest SR, comparable to native species. This discrepancy was particularly noteworthy as *A. pictum* shared closer phylogenetic relationships with European species, while *A. davidii* was more closely related to North American species. The observed phylogenetic signal in SR patterns suggests that intrinsic traits that relate to climate tolerance may be conserved yet masked in the conventional modelling approach. This interdisciplinary approach bridges the gap between macro‐scale predictions and local‐scale individual performance, offering a new perspective on niche conservatism through a phylogenetic framework.

## Graphical abstract

We investigated the relationship between ecological niche similarity and habitat suitability (HS) and between HS and standardised survival rates (SSRs) on the selected ex‐situ Acer species, A. davidii, *A. palmatum* , and *A. pictum* , in the UK. We also aimed to explain these relationships in a phylogenetic framework. We hypothesised that species with high niche similarities to native UK species will exhibit high HS, which in turn will correlate with high SR.

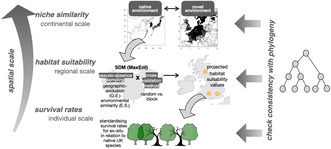

## 1 Introduction

Climate change has shifted the focus of ecological studies from ‘explanatory’ to ‘anticipatory predictions’ (Mouquet et al. 2015). The increasing need to predict the potential impacts of climate change on species' distribution patterns to support informed conservation decisions and risk assessment has propelled the popularity of the correlative species distribution models (Pearson and Dawson 2003). Essentially, species distributions in the area of interest are projected as habitat suitability (HS), or the probability of occurrence. These projections are derived by comparing environmental conditions at unsampled sites to those at sampled sites where species are present or absent (Elith and Leathwick 2009). Here, the presence–absence data serve as a proxy indicator of species' demographic performance at population level. The areas where species frequently occur are assumed to represent the environmental conditions that support ‘non‐negative population growth’, aligning with the Hutchinson's concept of ecological niche (Carscadden et al. 2020; Hutchinson 1957). This is intuitive because any observed presence is a manifestation of individuals that (1) achieved reproductive success and (2) survived local environmental conditions. The assumption that species' geographical range reflects its ecological niche easily leads to the idea that environmental conditions are most suitable–and thus population most abundant–near the range centre and decline towards the periphery, as suggested by the ‘abundant‐centre hypothesis’ (Brown 1984; Pironon et al. 2017).

However, studies show mixed evidence in relationship between abundance and geographic distance to range centre (Dallas et al. 2017; Jiménez‐Valverde 2012; Sagarin and Gaines 2002). This is largely due to non‐climatic factors (Canham and Thomas 2010). Demographic performance depends on diverse biological traits such as vital rates, dispersal ability, or genetic diversity (Bahn et al. 2006; Pagel et al. 2020; Pironon et al. 2017). The accuracy of species distribution can be improved by integrating these parameters directly or through surrogate measures, for example, phenological data (Canham and Murphy 2016; Chuine and Beaubien 2001; Morin et al. 2007; Pagel et al. 2020). Further, species distribution is ultimately conditioned, directly or indirectly, by the complex biotic interactions, which are challenging to quantify and often remain as unknown x (Novak et al. 2011).

The complex interplay of multiple ecological processes underlying species distributions creates ambiguity in discerning species' true optimal niche and their **ecological marginalities**. In particular, **geographic marginality** within a species' range should not be equated with **ecological marginality**. It suggests that a species' native distribution has limited utility in assessing **climate tolerance**, unless it is assessed independently of these interacting processes. As observed by Early and Sax (2014), invasive species could occur outside their native climatic range. Similar counter‐intuitive or unexpected responses to climate change are not uncommon in a real‐world and often exhibit highly variable interspecific magnitude (Parmesan and Hanley 2015). However, it is noted that these non‐conforming variations may still be addressed within conventional niche framework if properly tested (Clark et al. 2011; Wiens et al. 2010). For example, geographically separated species of shared ancestry can exhibit a certain degree of **niche conservatism**, even when the disjunct climatic conditions they experience differ in patterns (Qian and Ricklefs 2004). By incorporating phylogenetic relationships, seemingly independent event, such as species' survival beyond their climatic range, can be better understood. Yet, it remains uncertain how this subtle difference can be reflected in the modelled estimates, such as HS, and how they should be interpreted given increased complexity.

Thus, this research aims to analyse species' **climate tolerance**, by examining relationship between **survival rate (SR)** and HS, while incorporating phylogenetic relationships to provide more holistic understanding of species' response patterns. For this analysis, we focused on species located at their marginal niche, where SR > 0, outside their native geographic range. Although this approach may be best achieved in full scale, transplanting species across their entire environmental gradient, we limited our test to local botanic gardens for feasibility reasons. We considered that ex‐situ species in botanic gardens can serve as a valuable resource to test this concept. Botanic gardens provide a semi‐controlled environment that minimises certain biotic interactions (e.g., competition) found in natural settings, while still maintaining exposure to regional environmental conditions. Such settings may also limit SR assessment to a specific study period while omitting the long‐term biotic processes discussed earlier. However, the advantage is to assess species‐specific responses at the individual level, which will allow uncovering patterns in their relations to the recent climate change that might otherwise be obscured. Studying plants at the individual level, rather than using aggregated species data, proves particularly valuable when investigating species' responses to specific environmental changes, as it preserves critical information that would typically be lost when data are averaged across populations (Clark et al. 2011). Additionally, botanic gardens maintain long‐term inventory records that are well‐suited for comparative analysis with niche models at a matching temporal scale.

We quantified the degree of **niche marginality** for these ex‐situ species in relation to local native species of the same genus, rather than measuring their absolute distances from their native ranges. This approach uses locally abundant congeneric species—which are presumed to occupy within the optimal range of their ecological niche—as reference points, allowing us to test **niche conservatism** and to compare niche overlaps under phylogenetic relationships. We hypothesise that ex‐situ species with greater niche overlap with native UK/European species will show higher HS, with potential indication of **niche conservatism**. Furthermore, we expect these predicted HS will then correlate with species' SR relative to that of local species or **standardised survival rate (SSR)**. Such HS‐SSR agreement would confirm that the model effectively predicts thresholds for species' **climate tolerance**. Lastly, we aim to validate whether the alignment of niche similarity, HS, and SSR by species indicates any trace of **phylogenetic signal**—that is, the tendency for closely related species to share similar characteristics (Pearman et al. 2008), using the reference phylogeny map from Li et al. (2019).

**Glossary**

**Climate tolerance:** the ability of species to maintain viable populations through survival, growth, and reproduction under varying climatic conditions.

**Ecological marginality:** the degree to which a population exists near the limits of its species' physiological tolerance, determined by both abiotic and biotic conditions.

**Geographic marginality:** the degree of spatial isolation and fragmentation of populations relative to the core of the species' range, often associated with reduced connectivity and gene flow between populations.

**Habitat suitability (HS):** the capacity of an environment to provide the essential resources and conditions required for a species' survival, growth, and reproduction, which often translates to the probability of species’ occurrence.

**Niche conservatism:** the tendency for species of shared ancestry to retain similar fundamental and/or realised niches.

**Niche marginality:** a measure of the ecological distance between the species’ ecological niche and the available environmental conditions in the study areas.

**Niche similarity:** a degree of overlap or shared environmental conditions between the ecological niches of different species.

**Phylogenetic signal:** the tendency for closely related species to resemble each other more than they resemble distant relatives in their traits or ecological characteristics.

**Survival rate (SR):** The proportion of intraspecific individuals that survived relative to the initial population size within specified study areas.

**Standardised survival rate (SSR):** A measure of relative survival that represents how far an ex‐situ species' survival rate deviates from the mean survival rate of UK native species.

## 2 Methods

### 2.1 Outline of Research Processes

**FIGURE 1:**
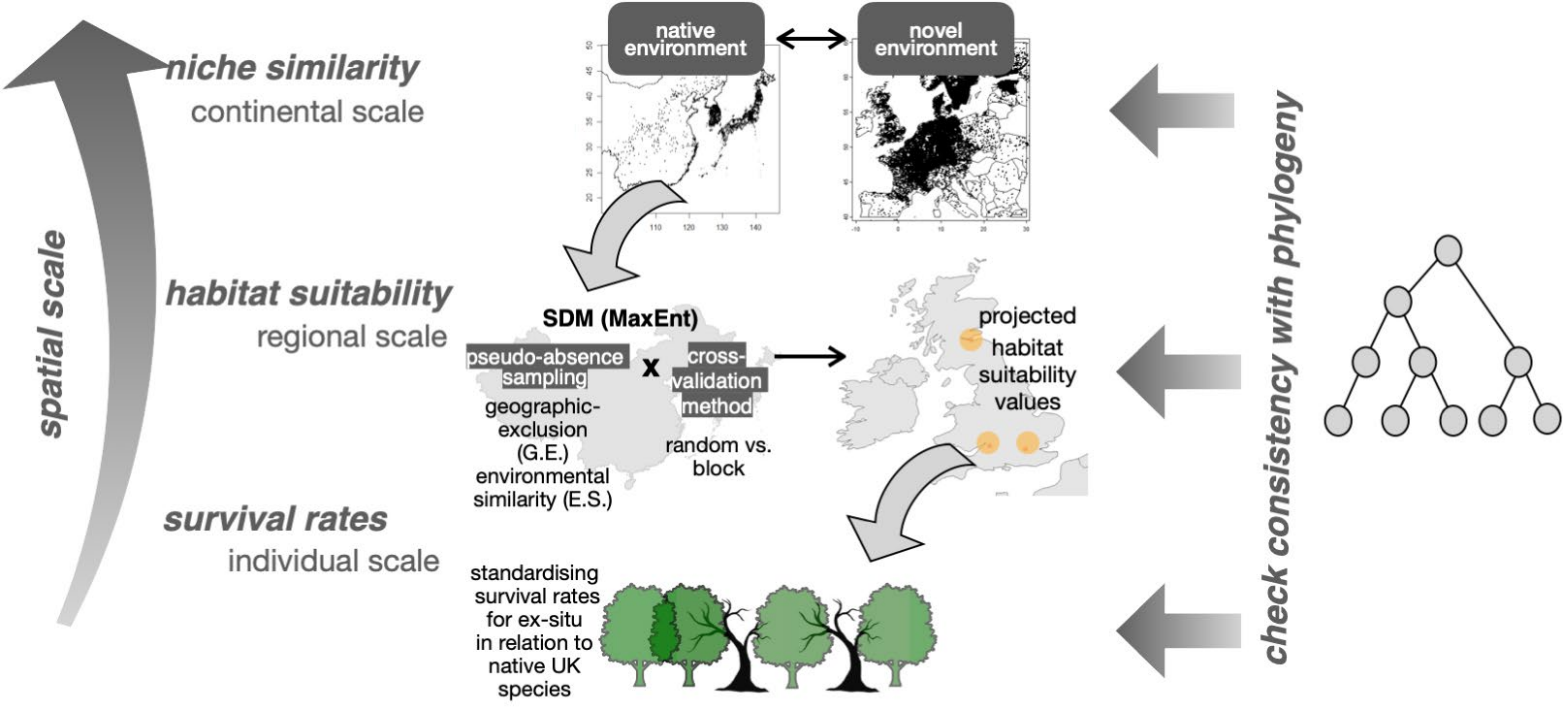
An overview of the analytical steps taken to capture processes acting at different spatial scales.

The study is structured into three main steps at different spatial scales, as illustrated in Figure 1. The first step involves evaluating the environmental similarities between species' native distributions and novel environments at a continental scale, using various selections of environmental variables. The second step involves projecting HS values for the three selected regions in the UK, from the trained model from their native range based on various combinations of configurations, specifically on pseudo‐absence sampling and cross‐validation methods. Lastly, the SR values from the previous research (Kim et al. 2023) are calculated and standardised to compare with modelled HS values at an individual scale.

### 2.2 Species Occurrence Data

**FIGURE 2:**
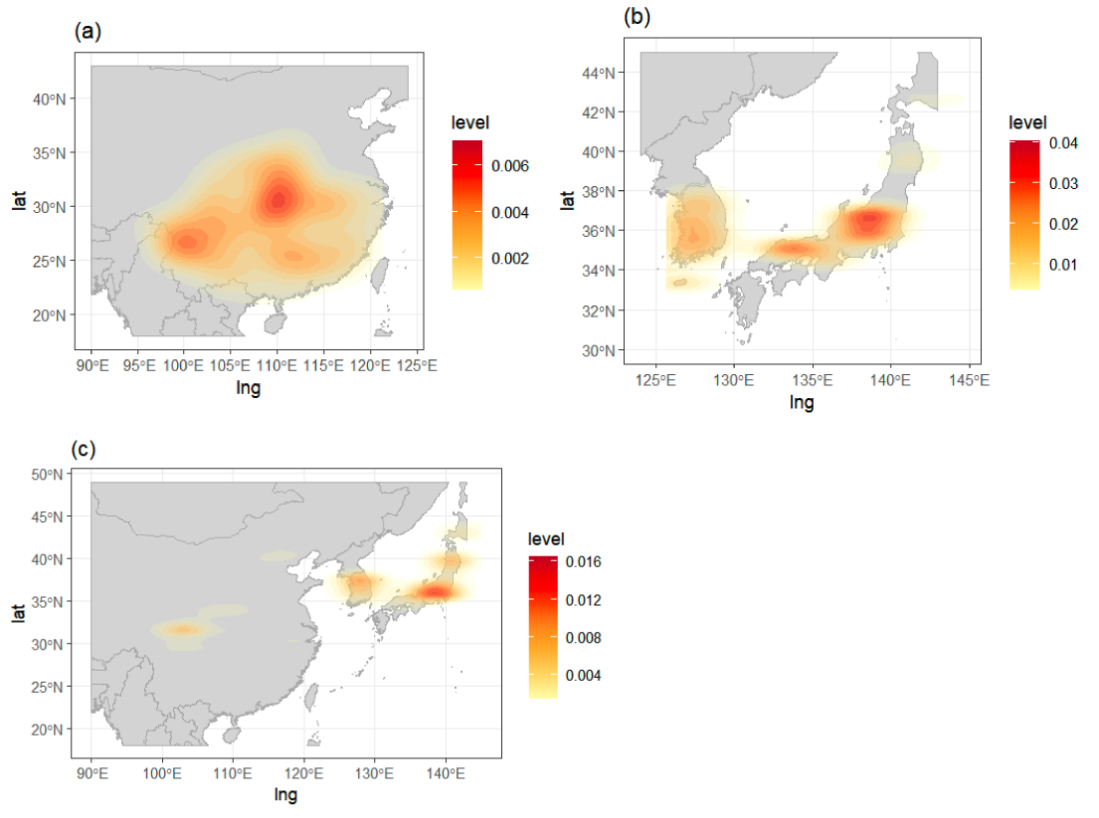
Occurrence density of (a) *A. davidii* (*n* = 222), (b) *A. palmatum* (*n* = 925), and (c) *A. pictum* (*n* = 2671) in their native range. Two‐dimensional kernel density plots of occurrences are overlaid on the map of each study region.

Three *Acer* species were selected, *A. davidii*, *A. palmatum* , and *A. pictum* , as each species characterises a distinct geographical range (Figure 2). Occurrences of *A. davidii* are primarily concentrated in Sichuan, extending to Southern China, which ranges from subtropical zones to cooler montane areas (Su et al. 2021). In contrast, *A. palmatum* is more prominently distributed throughout the southwest of Korea and from Honshu to Kyushu in Japan (Chang 1990). *A. pictum* exhibits a broader distribution, predominantly in high‐latitude cool temperate forests across all three countries.

All occurrence data were retrieved from the Global Biodiversity Information Facility (http://www.gbif.org; GBIF, 2024; see Supplemntary File S3 for citations for individual data download) and filtered for invalid occurrences (e.g., non‐terrestrial) using the CoordinateCleaner R package v2.0–20 (Zizka et al. 2019). From the downloaded data, only those sources of natural distributions such as herbariums or national surveys were selected, and any occurrences outside known native ranges, referenced from the Plants of the World Online (POWO: https://powo.science.kew.org), were excluded. Among the selected species, *A. pictum* and *A. davidii* posed a unique challenge regarding potential sampling bias as presence‐only data (Elith et al. 2011; Sillero et al. 2021). While these species are widely distributed across the studied region, imbalanced sample sizes between countries could result in over‐clustering in certain countries while underrepresenting their distribution in China, despite China's dominant geographical extent in East Asia. To minimise disparity among countries, we selected only national‐scale survey datasets from relatively data‐abundant countries, filtered by institution code. For example, institution code ‘BDCJ’ is the 2nd and the 3rd national survey datasets for *A. pictum* in Japan. The complete list of specific datasets selected for each country is available in Supplemental File III. Additionally, all data were rarefied to the resolution of environmental variables so that a minimum 1 km distance between occurrences were maintained using the spThin R package v0.2.0 (Aiello‐Lammens et al. 2015).

### 2.3 Environmental Variables

The choice of environmental variables that adequately capture species distribution constraints is critical for developing reliable models. Moreover, selecting environmental variables that directly influence on physiological responses would be ideal but challenging when relating modelled projections to local SRs (Gardner et al. 2019; Guisan and Zimmermann 2000). Therefore, we ran models with different combinations of climatic, soil, and land cover variables derived from multiple global databases (Table S1) to evaluate their relative performance. As climate variables are most critical and diverse, they are categorised into three groups by their distinct influence on plant physiology, ‘bio A', ‘bio B' and ‘bio C'. The first group, ‘bio A‘, was chosen from a pool of 19 common bioclimatic variables that characterise broad and general climate. The second group, ‘bio B', was chosen from a pool of all 19 and additional CHELSA‐BIOCLIM+ variables (Brun et al. 2022), which were processed to characterise growing season conditions, specifically including water balance, solar energy and evapotranspiration demand. The last group, ‘bio C', consists of variables that reflect specific seasonal effects and thresholds linked to biophysical tolerance such as minimum temperature of the coldest month. The selections of ‘bio A' and ‘bio B' were determined based on a paired correlation test, retaining only variables with its correlation coefficients < 0.70 (Dormann et al. 2013), while ‘bio C' was manually selected. The specific list of variables for each group is detailed in supplemental Table S1. All bioclimatic data were obtained from CHELSA v2.1 climatologies at a spatial resolution of 30 arc sec (∼1 km at the equator) (Brun et al. 2022; Karger et al. 2021). All correlation tests were performed using the ‘caret’ package (Kuhn et al. 2023).

For soil variables, selections were made based on their ecological relevance to nutrient availability and water‐holding capacity (Dodd and Lauenroth 1997; Eyre 2013). Global soil profile data, including soil acidity (pH), clay, silt, and bulk density of the upper soil layer (0–5 cm), were downloaded from SoilGrids 2.0 (Poggio et al. 2021) at a resolution of ~250 m and subsequently resampled to ~1 km. Among them, sand content was excluded due to its strong correlation with clay and silt content. Lastly, remote‐sensed land surface characteristics, such as enhanced vegetation index (EVI) and EVI dissimilarity index derived from the Moderate Resolution Imaging Spectroradiometer (MODIS), were download from the EarthEnv (Tuanmu and Jetz 2015) at a spatial resolution of 30 arc sec (~1 km at the equator). These vegetation indices are indirectly influencing ecosystem attributes such as soil moisture, land surface temperature, and intercepted light amount (Regos et al. 2022). Given that the selected *Acer* species are generally known to be shade‐tolerant subcanopy species, we included these vegetation indices to test if these variables would improve our models. However, both EVI and EVI dissimilarity indices are heavily dependent on temporal factors, making it crucial to establish the appropriate temporal condition. In this study, annual EVI dissimilarity, EVI*dis*, and the maximum monthly EVI, EVI*max*, for each year were averaged across the preset period from 2001 to 2005. All downloaded environmental variables were adjusted to a resolution of 30 arc sec and stacked to align on the same grid.

### 2.4 Niche Similarity and Phylogeny

Niche similarity test was performed to evaluate environmental similarities between the native distributions of the ex‐situ species selected in 2.2 and the UK/European distributions of native species (Broennimann et al. 2012; Warren et al. 2008). We selected two widely distributed *Acer* species in the UK, *A. campestre* and *A. platanoides* (Figure S1) for the comparison. It is noted that *A. platanoides* is not strictly native to the UK but is extensively naturalised across the UK, comparable to ‘near‐native’. The niche similarity test evaluates the niche overlap between the two species based on their observed distributions in comparison to their background overlap based on the random points drawn from the broader range each species occurs. The null hypothesis—that the observed niche overlap does not differ from their random background overlap by chance—can be rejected if the *p*‐value is below 0.05 after 100 simulation runs. For each species pair, the test was conducted at two geographical scales: the UK range and the entire range in Europe. This dual approach allows us to determine whether the similarity test result is specific to the UK range or consistent with the full ecological range. This comparison is particularly important for interpreting **niche marginalities**, as these species' geographic range extend beyond the UK, covering broader European regions. This test is primarily based on the metrics, Schoener's D (Schoener 1970) and Warren's I (Warren et al. 2008). A score of one indicates complete overlap, while a score of zero indicates no overlap. All tests were conducted using ‘ENMTools’ v1.1.1 (Warren et al. 2021).

In addition to the niche similarity test, phylogenetic relationships were examined using reference genome analysis by Li et al. (2019). This is to evaluate the link between niche similarity and species' phylogenetic relatedness.

### 2.5 Species Distribution Modelling

Among the various modelling methods available, MaxEnt v3.4.4 (Phillips and Dudık 2008) was chosen for its demonstrated transferability with presence‐only data (Elith et al. 2006; Heikkinen et al. 2012). MaxEnt also shows relatively higher sensitivity compared to other modelling methods, which has the advantage of identifying potential niches beyond the calibrated range (Barbet‐Massin et al. 2012; Qiao et al. 2019). In this study, species' realised niches were defined by their native distributions (training region), while UK regions were evaluated as their potential niche by projected HS. The underlying assumption is that anthropogenic introduction of these species to UK has conceptually removed dispersal barriers, with projected HS representing the hypothetical approximation for species' realised niches to expand towards their fundamental niches (Soberon and Peterson 2005).

#### 2.5.1 Preprocessing Training Region

Prior to modelling, we delineated the training regions for each selected species by generating polygons around each occurrence with a buffer radius of 200 km. The distance was determined based on the reference study demonstrating optimal performance of areas under the receiver operating characteristic curve (AUC) (Wal et al. 2009).

#### 2.5.2 Pseudo‐Absence Sampling Strategy

In addition to occurrence data, pseudo‐absence sampling strategy also equally influences the model's predictive accuracy. To minimise the negative effects of false pseudo‐absences, we employed (1) the ‘geographic‐exclusion (*G.E*.)’ approach (Barbet‐Massin et al. 2012) and (2) the ‘environmental similarity (*E.S*.)’‐based approach (Chefaoui and Lobo 2008; Wang et al. 2023). The ‘*G.E*.’ approach is based on the simple assumption that any region within a certain minimum distance from an observed occurrence shares similar environmental conditions and is considered suitable. We thus created a smaller buffer of either 10 km (for *A. palmatum* ) or 30 km (*A. davidii* and *A. pictum* ) around occurrences to avoid generation of pseudo‐absences in close proximity to known occurrences. The distance was determined based on the spatial extent, the number of occurrences, and buffering distances used in comparable studies (Barbet‐Massin et al. 2012; Chefaoui and Lobo 2008; Wang et al. 2023). For the ‘*E.S*.’ approach, unsuitable areas for pseudo‐absence generation were preliminarily defined based on environmental similarity using One‐Class Support Vector Machine (OCSVM), a popular machine learning algorithm for anomaly‐detection (Senay et al. 2013). A set of pseudo‐absences was generated from the resulting unsuitable areas for each set of environmental variables selected in 2.2. The number of pseudo‐absences generated were 10,000 for *A. palmatum* and *A. pictum*, and 5000 for *A. davidii* to balance with a lower count of observed occurrences. This random sampling was repeated 5 times.

#### 2.5.3 Cross‐Validation Strategy

For this study, it is important to first validate species distribution models within the species' native region. By establishing the model's reliability in their known region, we can then assess how consistently the well‐trained model's HS predictions align with the actual SSR in the novel environment. Ideally, model validation requires the independence of the test data, that is, test data must not relate to training data. For example, it is recommended to use occurrence data from the geographically distinct subsets of the native regions to avoid spatial autocorrelation. When test data is randomly extracted from the training region where spatial autocorrelation exists, the test and train data share similar patterns by increased chance of pulling test data from those in geographic proximity to train data. Because this risks inflating the model's reliability with over‐optimistic confidence, the study compares and contrasts two cross‐validation strategies: (1) a geographic block‐partitioning strategy (‘*block*’) and (2) a random partition for comparison (‘*random*’) (Roberts et al. 2017). The former, ‘*block*’ approach allows a self‐extrapolation test within the native range by leaving out all occurrences within selected geographic blocks for testing. The latter, ‘*random*’ approach uses test data randomly selected from the entire studied region, regardless of geographic proximity. By comparing validation results from these two approaches, we can compare the potential predictive errors when transferring the trained model to novel environments.

For the ‘*block*’ strategy, spatial blocks were generated and assigned to study regions in folds (*k* = 4), allowing even allocation of occurrence data across specified folds. The training data are taken from *k*‐1 folds while the remaining data are used in validation. The size of the square blocks for each species was automatically determined using the ‘cv_spatial_autocor’ function from the BLOCKCV library v3.1–3 (Valavi et al. 2023). For the ‘*random*’ strategy, 25% of occurrence data were set aside for cross‐validation. For both approaches, 10‐fold cross‐validations paired with 5 sets of pseudo‐absence samples resulted in a total of 50 models.

The performance of the model was evaluated using two metrics: the True Skill Statistics (TSS) that combine sensitivity (true positive rate) and specificity (true negative rate) into a single metric ranging from −1 to +1, and the AUC that discriminates between presence and absence across all possible threshold values (0–1).

#### 2.5.4 Projecting Habitat Suitability (HS)

All trained models from native regions were projected onto the UK region to generate HS. These resulting HS values were then averaged to create a single projection. The raw HS values were multiplied by 0.01 to standardise from highest suitability, 1, to lowest suitability, 0. Lastly, HS values assigned to each cell were aggregated and generated a single mean value for each local district corresponding to the location of selected urban botanic gardens. Specifically, these local districts are Richmond upon Thames for KEW, Cotswold for WESB, and Midlothian and West Lothian for RBGE. For Edinburgh, surrounding counties were selected rather than the city itself, as soil variables were only available for non‐urban areas.

The complete modelling process illustrated from 2.5.1 to 2.5.4 was performed using ‘dismo’ v1.3–9 (Hijmans et al. 2017) and ‘biomod2’ v4.2–4 (Thuiller et al. 2023) and run on the high‐performance cluster at the University of Sheffield.

### 2.6 Standardising Survival Rates (SSRs)

Mortality rates for the selected species were obtained based on the previous research (Kim et al. 2023). These retrieved mortality rates were originally collected from the three botanic gardens in the UK; the Royal Botanic Gardens Kew (KEW), Westonbirt, the National Arboretum (WESB), and the Royal Botanic Garden Edinburgh (RBGE). Specific methods used in filtering inventory data and calculating the mortality rates can be found in the original paper. Briefly to summarise, all mortality events were filtered out to leave only those explicitly related to climate or unknown cause. The retrieved mortality rates were then converted into species‐specific SR and standardised as follows,

$${\mathrm{SR}}_{\mathrm{ijk}}=1-{\mathrm{MR}}_{\mathrm{ijk}}$$

$${\mathrm{SSR}}_{\mathrm{ijk}}=\frac{\left({\mathrm{SR}}_{\mathrm{ijk}}-{\mathrm{SR}}_{\mathrm{\mu jk}}\right)}{\sigma }$$

where SR for species *i* in a given period *j* (2000 to 2021), within region *k* was first calculated from the species‐specific mortality rate, MR. The resulting SR is then standardised in relation to the native species' mean SR, ${\mathrm{SR}}_{\mu }$, with its standard deviation, σ. The purpose of SSR is to calculate relative distance to mean SR, ${\mathrm{SR}}_{\mu }$ of locally native species.

**FIGURE 3:**
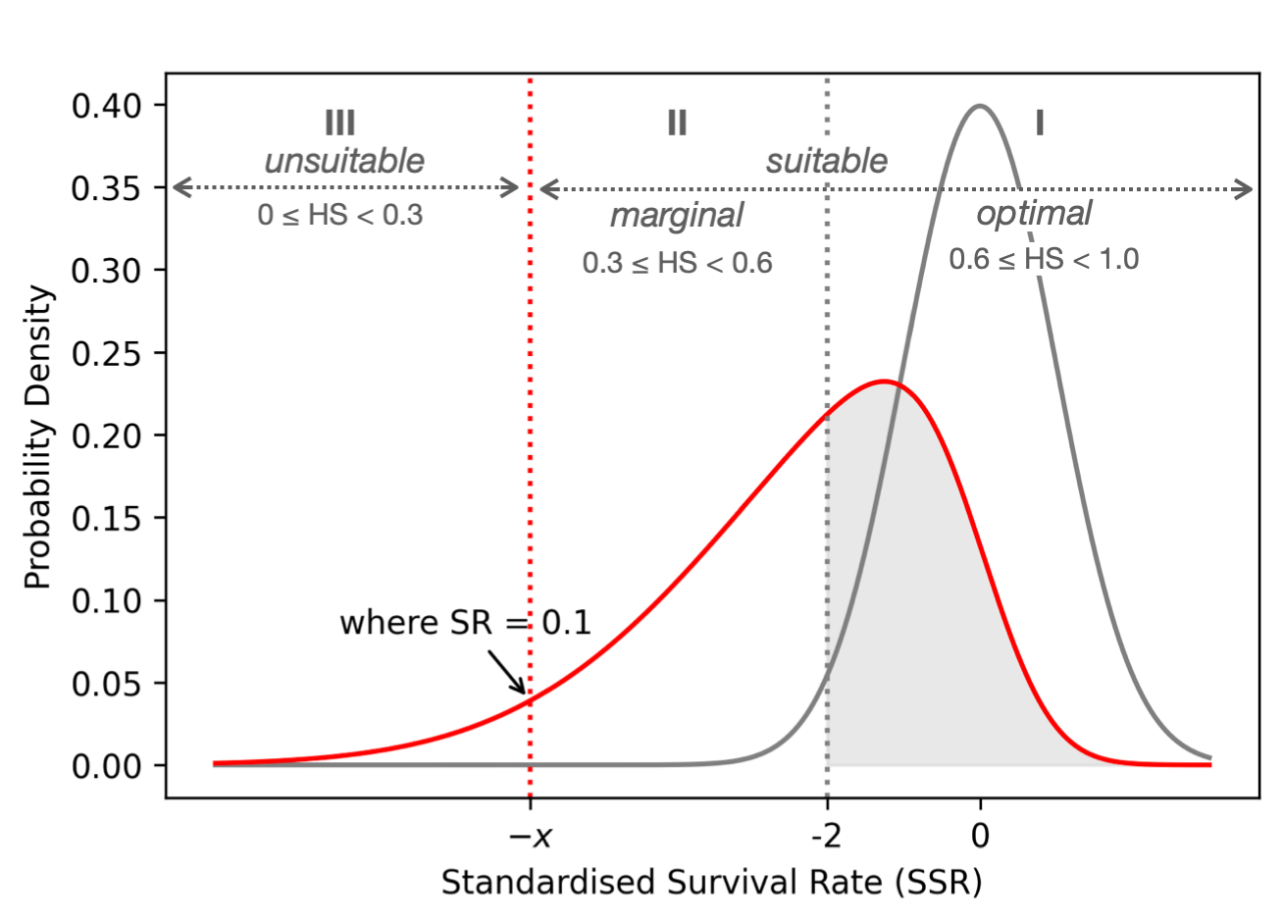
A hypothetical distribution curve for ex‐situ species' standardised survival rates (SSR) shown in red, in relation to native species' SR distribution shown in grey. Three intervals of habitat suitability (HS) values are mapped to SSR: The optimal interval I, HS between 0.6 and 1.0, is mapped to the 95% SR range of native species; the unsuitable interval III, HS between 0 and 0.3, corresponds to areas below where ex‐situ species' SR < 0.1; the marginal interval II represents the range between the unsuitable and the optimal interval.

The analysis first assumes that the SRs of native species follow a normal distribution. For region *k*, SSR values within ±2 indicate that species' SR falls within the expected range of native species with 95% confidence. This ±2 window thus defines the ‘suitable’ range, where species' performance is comparable to that of locally established species. However, when ex‐situ species show increasingly higher mortality rates, the distribution becomes left‐skewed (Figure 3). In such a left‐skewed curve, the lower bound of the 95% confidence interval can hypothetically shift towards the left, reaching a minimal SR of 0.1, denoted as −χ. Consequentially, the suitable range that is comparable to native species narrows, while a larger proportion of SRs falls below this suitable range but remains just above the minimal SR (> 0.1). This intermediate range is considered marginal but still suitable, as species are surviving but not thriving. It is important to distinguish this marginally suitable range from the optimally suitable range, especially when assigning corresponding HS values.

HS values are typically continuous, ranging from 0 to 1. Converting these values into binary presence/absence predictions requires setting an arbitrary threshold, often at 0.5 (Li et al. 1997). For presence‐background algorithms such as MaxEnt, the threshold can be lowered to 0.3 or even below in certain cases, as these algorithms calibrate based on a large pseudo‐absence samples across broad background areas (Jiménez‐Valverde and Lobo 2007; Sillero et al. 2021). We set a threshold of 0.3. Areas below this threshold are considered ‘unsuitable,’ corresponding to locations where SR is predicted to fall below 10%. Areas above this threshold can be more ambiguous in relating to SR as species presence alone does not necessarily indicate demographic performance (Roloff and Kernohan 1999). However, a midpoint threshold of 0.6 was arbitrarily applied to differentiate between marginally suitable and optimally suitable locations.

Lastly, in addition to their relation to HS, all species‐specific SRs were examined in relation to their phylogeny to identify any consistent patterns among more closely related species. For this analysis, the SRs of species identified as sister species were rearranged based on the previous research (Kim et al. 2023).

## 3 Results

### 3.1 Niche Similarity and Phylogeny

Overall, *A. davidii* demonstrated greater niche overlap with both *A. campestre* and *A. platanoides* , with this overlap particularly significant within the UK distributions compared to the entire distributions across Europe (Table S2). This contrasts with *A. palmatum* and *A. pictum* , which exhibited significantly greater niche similarity with *A. platanoides* across broader Europe, but not within the UK (Table S3). It is also noted that *A. palmatum*, in particular, exhibited no overlap, a very strong dissimilar climatic pattern within the UK range.

According to the phylogenomic analysis conducted by Li et al. (2019), each species belongs to a different section: *A. davidii* to section *Macrantha*, *A. palmatum* to section *Palmata*, and *A. pictum* to section *Platanoidea*. Interestingly, both of the UK native species selected for niche comparison belonged to the section *Platanoidea*. Based on this phylogenetic tree, *A. pictum* is most closely related to these UK species, while *A. palmatum* is most distantly related.

### 3.2 Comparing Model Predictions Between Native and Novel Environment

**FIGURE 4:**
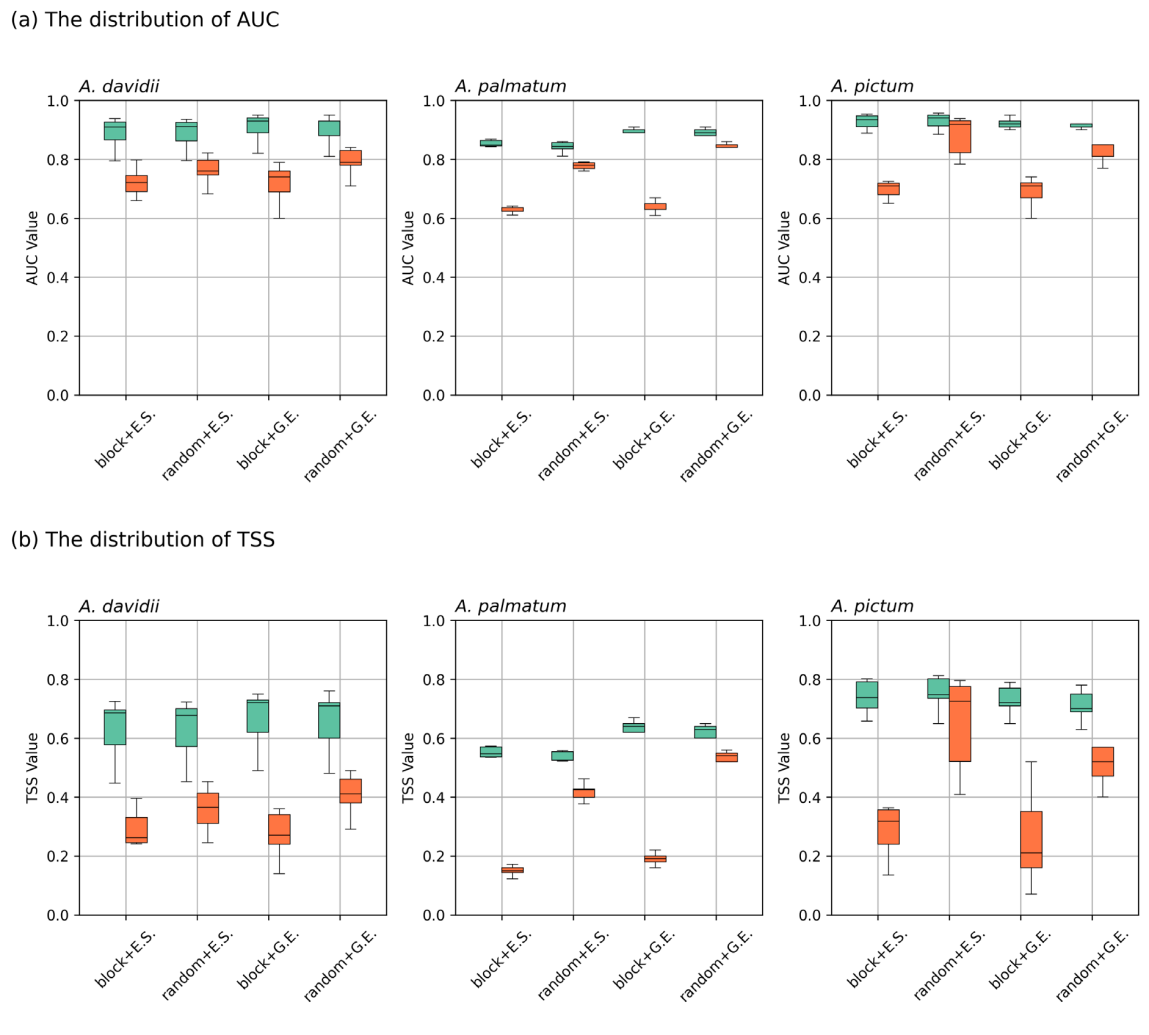
The comparative distributions of model evaluation output. Model performance varies with different combinations of cross‐validation and pseudo‐absence strategy for each species, *A. davidii, A. palmatum*, and *A. pictum*. (a) AUC with calibrated means (green) and validated means (red), and (b) TSS with calibrated means (green) and validated means (red).

**FIGURE 5:**
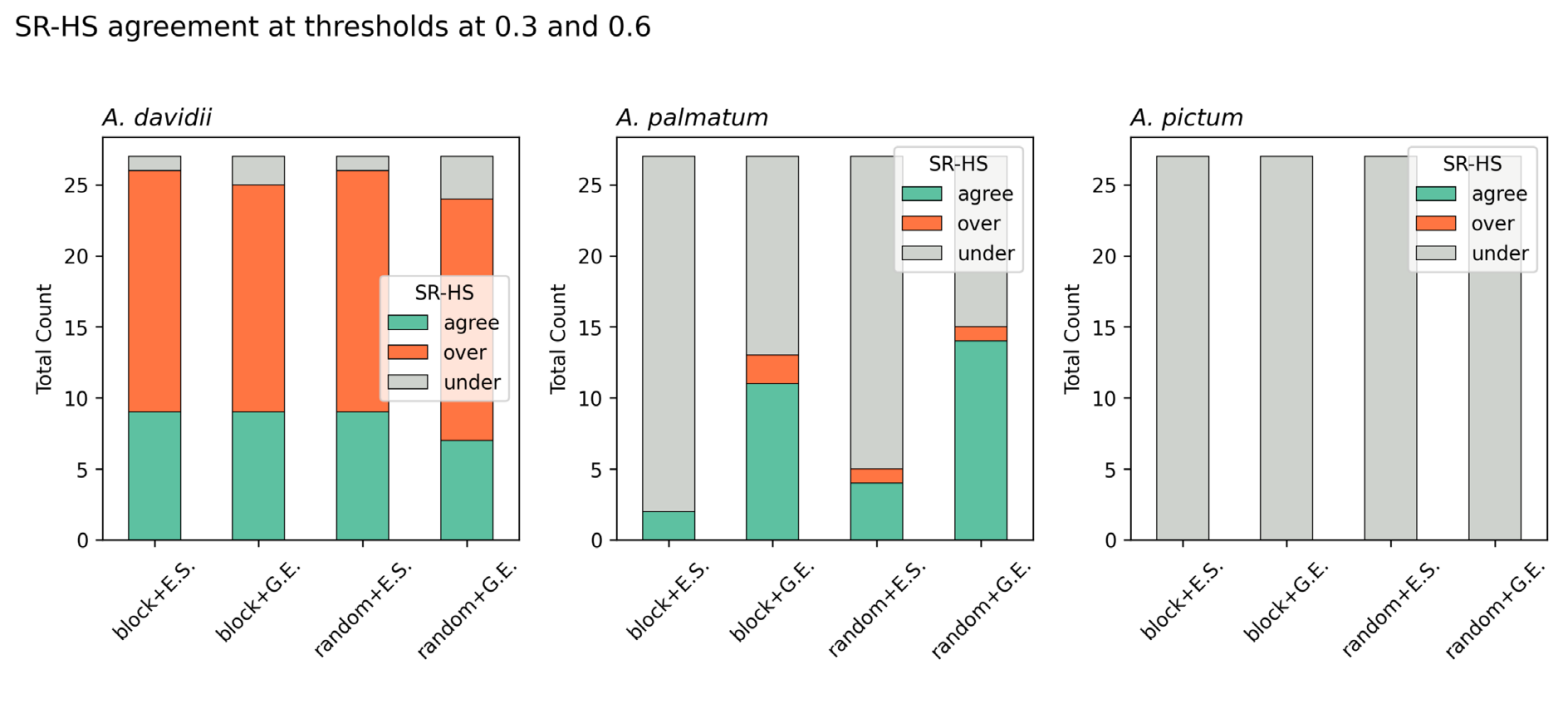
The cumulative count of alignment between the predicted habitat suitability (HS) and survival rate (SR) from all models for species, *A. davidii, A. palmatum, and A. pictum*. The alignment between HS and SR is determined by the mapping illustrated in Figure 3 as HS = SR indicated by ‘agree’ in green, HS>SR indicated by ‘over’ in orange and HS<SR indicated by ‘under’ in grey. The threshold 0.3 classifies unsuitable‐suitable while the threshold 0.6 classifies marginal‐suitable. Pseudo‐absence sampling based on environmental similarity is abbreviated as *E.S* and the geographic exclusion strategy is abbreviated as *G.E*.

Within species' native range, the overall discriminatory power, measured by AUC, was fairly good with mean validation values of 0.74, 0.72, and 0.78 for *A. davidii*, *A. palmatum* , and *A. pictum* , respectively. In contrast, the model's accuracy, measured by TSS, decreased sharply from moderate to weak, with its mean validation values of 0.33, 0.32, and 0.44 for *A. davidii*, *A. palmatum* , and *A. pictum* , respectively. As discussed in 2.5.3, the *random* validation approach generally produces higher TSS scores than the *block* validation approach (Figure 4). These results suggest model prediction capacity weakens when validated using independently held‐out block, which represents novel environment outside the training range. Furthermore, the choice of validation approach did not substantially affect the prediction alignment between HS and SR for *A. davidii* (Figure 5). However, the choice of pseudo‐absence sampling strategy had a stronger influence on presence–absence predictions for all species. When comparing only pseudo‐absence sampling approaches while keeping all other configurations set constant including environmental variables, the model using the ‘*G.E*.’ approach improved the model performance, yielding higher TSS scores. For *A. palmatum* , the best model combined *random* validation with the *G.E*. pseudo‐absence sampling approach (‘*random + G.E*.’), using either climate‐only ‘bio A' (TSS 0.56) or ‘bio B' (TSS 0.52) variable selections (Supplemental file I). These were only models that achieved both the highest validation TSS scores and perfect agreement between HS‐SR in all three sites. For *A. davidii*, the best performing models were the ‘*random + G.E*.’ approach using the complete bio A variable set that includes soil and land cover (TSS 0.46) and the ‘*random + E.S*.’ approach using the complete bio B variable set (TSS 0.45). However, while these models demonstrated good HS‐SR alignment (two out of three aligned), it should be noted that the overall degree of HS‐SR alignment resulted similar among different model configurations for *A. davidii*. Furthermore, none of the resulting estimates for *A. pictum* showed any agreement between HS‐SR except one model using ‘*block + E.S*.’ approach that predicted two regions–Lothian and Richmond–marginally suitable, with a full bio C variable set (TSS 0.25). This misalignment in particular contrasts with *A. pictum* 's high prediction capacity shown in its native range, exhibiting highest model performance among the studied species.

Overall, additions of soil and land cover variables improved model performance for *A. davidii* and *A. pictum* at least for binary presence–absence predictions within their native range. This contrasts with models for *A. palmatum*, which performed better without soil and land cover variables: it exhibited the best predictions in species' native range with ‘bio C' (highest TSS) and the highest alignments between HS‐SR and ‘bio A'.

### 3.3 SR‐HS Alignment Within Phylogeny

**FIGURE 6:**
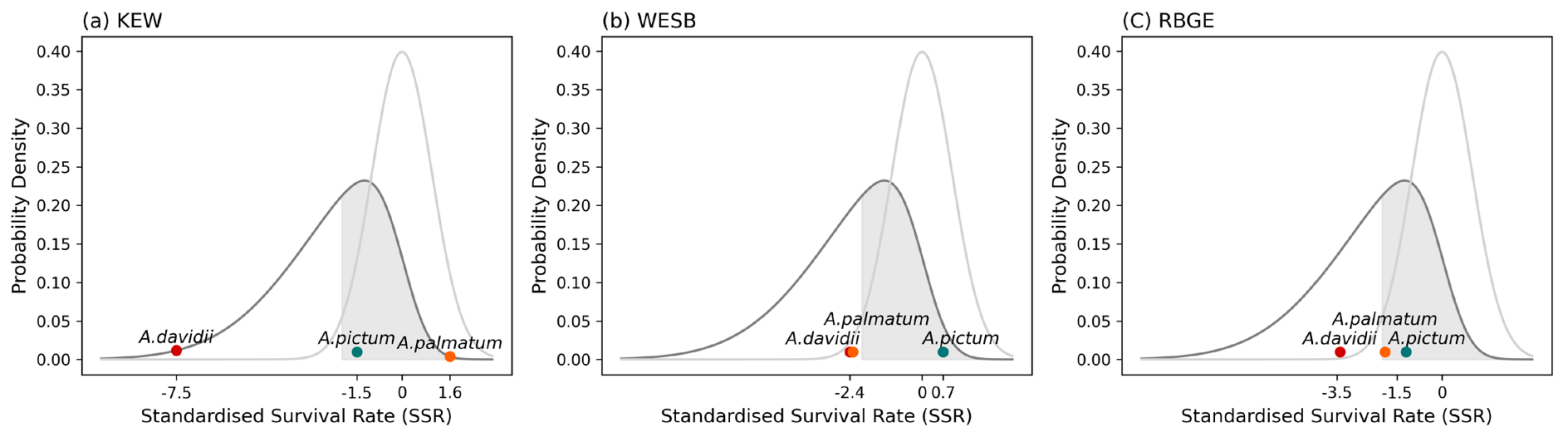
Relative position of Standardised Survival Rate (SSR) for *A. davidii* (red), *A. palmatum* (orange), and *A. pictum* (green) in each botanic garden: (a) Royal Botanic Gardens, Kew (KEW) (b) the National Arboretum, Westonbirt (WESB), and (c) the Royal Botanic Garden Edinburgh (RBGE).

While the aggregated means of projected HS, collapsing regional differences, were highest in the order of *A. davidii* (0.66) > *A. palmatum* (0.40) > *A. pictum* (0.09), the SR followed exactly the reverse order, with aggregated SR means of 0.53, 0.73, and 0.81, respectively. This means that some models for *A. davidii* showed moderate agreements between the projected HS and SR, but many other models yielded overestimated values (Figure 5). In contrast, the projected HS of the other two species was mostly underestimated. Contrary to optimistic HS estimation, *A. davidii* exhibited the lowest range of SRs, all below 0.6, with the lowest value of 0.49 observed at KEW (Richmond). When standardised against native species, all SRs observed fell below the 5th percentile (SSR < −2) of native species' range (Figure 6). On the other hand, *A. pictum* demonstrated the highest SR among the selected species, with all values above 0.7, within a range comparable to that of the native species. The SR of *A. palmatum* varies by region, with the highest value of 0.87 observed at KEW (Richmond), followed by 0.70 at RBGE (Lothian), and 0.61 at WESB (Cotswold), which falls slightly outside the native range (SSR −2.26). Native species in these regions show SRs ranging from 0.75 to 1.0, with regional means of 0.8, 0.88, and 0.87 for KEW (Richmond), RBGE (Lothian), and WESB (Cotswold), respectively.

Additionally, these SRs are compared among other *Acer* species within the phylogenetic group (Table 1). It is observed that section member species display similar SRs among themselves. For example, within sect. *Platanoidea*, to which *A. pictum* belongs, species such as *A. cappadocicum* (0.84 ~ 0.91), *A. platanoides* (0.75 ~ 0.85), and *A. campestre* (0.84 ~ 1.0) exhibit a similar range of high SRs. On the other hand, those member species within sect. *Macrantha*, to which *A. davidii* belongs, have shown relatively lower SRs as observed in *A. pensylvanicum* (0.29 ~ 0.59) and *A. crataegifolium* (0.25 ~ 0.43). Lastly, *A. palmatum* belonging to sect. *Palmata* also shows a similar range of SRs with member species within this section such as *A. japonicum* (0.73 ~ 1.0) and *A. circinatum* (0.71 ~ 0.86). In particular, sect. *Macrantha* statistically differed from the rest of the species, with markedly lower ranges of SRs in all three sites. Additionally, patterns are not only evident in the interspecific SRs but also evidenced in the variations of intraspecific SRs among the botanic gardens. For instance, the SRs of those species from sect. *Macrantha* are similarly lowest at KEW and highest at WESB, whereas those species from sect. *Palmata* exhibited the opposite patterns, lowest at WESB and highest at KEW.

**TABLE 1:** Survival rates (SRs) by phylogenetic group and botanic garden. The italic numbers indicate SRs of the selected species (in bold text) in this study.

Phylogenetic group	Native to	Species	Survival rates by botanic garden		
			KEW—Richmond	RBGE—Lothian	WESB—Cotswold
**Sect. Macrantha**	Asia	*A. crataegifolium*	0.43	0.25	0.40
	**Asia**	***A. davidii***	*0.46*	*0.55*	*0.59*
	Asia	*A. rufinerve*	0.57	0.73	0.67
	America	*A. pensylvanicum*	0.29	0.48	0.59
	Asia	*A. tschonoskii*	NA	NA	0.62
	mean		0.44***	0.50*	0.57*
*Sect. Palmata*	Asia	*A. campbellii*	NA	0.76	0.75
	America	*A. circinatum*	NA	0.86	0.71
	Asia	*A. japonicum*	1.00	0.87	0.73
	**Asia**	***A. palmatum***	*0.87*	*0.70*	*0.61*
	mean		0.94	0.80	0.70
*Sect. Platanoidea*	Europe	*A. campestre*	0.84	1.00	1.00
	Europe	*A. platanoides*	0.75	0.86	0.85
	Asia	*A. cappadocicum*	0.91	0.84	0.86
	**Asia**	***A. pictum***	*0.73*	*0.70*	*0.95*
	mean		0.81	0.85	0.92

*Note:* The Mann–Whitney U test was conducted to compare each section against the other sections for each site. ***p < *0.001*,   *p < *0.05*. The section that showed a significant difference in SR distribution compared to other sections is highlighted, with its mean SR shown in bold.

## 4 Discussion

### 4.1 Phylogenetic and Environmental Distance Are Complementary Rather Than Collinear

*Acer* is a highly diverse genus, comprising approximately 152 species, with its ancestral biogeographic region disputed between Eastern Asia (Gao et al. 2020; Li et al. 2019) and North America (Wolfe and Tanai 1987). There are about a dozen species native to Europe, while the majority of *Acer* species are predominantly distributed in Eastern Asia. The selected ex‐situ species in this study—*A. davidii*, *A. palmatum* , and *A. pictum—each* originated from Eastern Asia, representing a distinct phylogenetic section: *Macrantha*, *Palmata*, and *Platanoidea*, respectively, with a distinct biogeographical range (Figure 2). Among the sections, *Macrantha* represents the earliest divergence, while *Palmata* is the latest divergence and contains the largest number of species (de Jong 1976; Li et al. 2019). Both sections consist of species with a disjunct distribution in East Asia and North America. In contrast, section *Platanoidea* consists of species from Europe and East Asia: *A. campestre* , *A. platanoides* , and *A. pictum* are all under one umbrella.

Therefore, our hypothesis test worked for *A. pictum* , which exhibited significantly greater niche similarity with its sister species, *A. platanoides* , despite their non‐overlapping geographic distributions. *A. pictum* and *A. platanoides* are indeed most closely related, with the shortest phylogenetic distance and very low genetic divergence observed (Suh et al. 2000). Both species are distributed at relatively higher latitudes, and their distributions are largely constrained by winter temperatures. Nonetheless, *A. pictum* did not show significant similarity to *A. campestre* , despite being a member species within the same section. As observed in these results, phylogenetic relationships and niche overlap are not always straightforward. Some studies found that niche similarity did not directly correlate with the phylogenetic distance, and niche differentiation could occur at any phylogenetic level (Knouft et al. 2006; Silvertown et al. 2001).

Essentially, phylogeny is more concerned with evolutionary patterns in shaping functional traits and how these traits are conserved within a lineage (Ackerly 2003). The combination of two pressures, (1) environmental filtering–selecting favourable traits–and (2) competitive interaction–differentiating traits–has led to either variability or conservation of these traits over time (Ackerly 2003; Thakur and Wright 2017). Therefore, the phylogeny‐environment relationship provides a complementary perspective rather than a linear covariate. For example, while closely related species conserve traits through environmental filtering, the same mechanism also drives traits to ‘converge’ and become similar when distant species need to co‐exist (Cavender‐Bares et al. 2004; Thakur and Wright 2017). Further, closely related species tend to diversify traits or habitat features due to competitive interaction over the limiting resource.

Considering the complexity between environment‐trait, the resulting niche similarity test based on macro‐environmental variables may not always accurately reflect the degree of conserved ecological traits. It is more likely that species show conserved traits when congeneric species show a certain degree of conserved niche (Warren et al. 2014), as may be the case with *A. palmatum* and *A. platanoides* in this study (Table S3). However, while low niche overlap between relative species initially indicates low niche conservation at the macro‐scale, these species may still share certain conserved traits depending on their biogeographic history and evolutionary patterns (Cavender‐Bares et al. 2004; Losos 2011).

Nonetheless, niche similarity tests yielded inconsistent results when tested on the different spatial scale—UK vs. Europe—or with different selections of variables. For example, *A. davidii*, which exhibited no significant niche overlap with native species across Europe, displayed significant niche overlap over the UK range. This contrasts with the other two species that exhibited significant niche similarity with native species across Europe but displayed no significant overlap over the UK range (Table S3). Notably, high HS predictions corresponded with significant niche overlap in the UK, whereas SRs generally corresponded with significant niche overlap at the continental scale. These findings suggest that niche similarity tests better reflect ecological traits when conducted across the full biogeographic range. However, establishing reliable connections between niche overlap and specific ecological traits requires further verification, as demonstrated here by using SRs as an indicator of climate tolerance.

### 4.2 Relating SR to Biogeographic Distributions

Our results demonstrated that models trained on species' native regions showed decreased predictive accuracy when applied to novel environments beyond the training regions. The decreased predictive accuracy was evident in a lower TSS score when using a *block* validation strategy within species' native range. When further applied to the UK environment, these models worked for at least two species in terms of binary predictions: they predicted most of the selected regions suitable for *A. davidii* and *A. palmatum* with HS well above the 0.3 threshold. However, interpreting HS on a continuous scale in relation to individual performance, discrepancies emerged. Specifically, models with strong predictability within their native range tended to overestimate (HS>SR) for *A. davidii* but underestimate (HS<SR) for *A. palmatum* and *A. pictum* .

However, these apparent over‐ and underestimations require careful interpretation. For example, the high HS values for *A. davidii* align with its significant niche overlap with native species in the UK range, suggesting that greater environmental similarity yields higher HS. While both the niche similarity test and trained models used the same climate data aggregated over the 1981–2010 period, the SR spans 2000–2021. This temporal mismatch, coupled with the intensifying climate change effects during the latter decade, may explain the observed discrepancy in HS‐SR. This is further evidenced by the increasing frequency of heatwaves, which have become annual events in Southeast UK from 2010 onwards, as documented by Kim et al. (2023). Moreover, mortality events of *A. davidii* at KEW reached a dramatic peak in 2021 (data not shown), reflecting accumulated heat and drought stress. Apparently, these drought stress effects were not incorporated into our models.

Other notable discrepancies between HS and SR were observed in *A. pictum* . While most regions were predicted to be unsuitable, empirical observations from botanic gardens in those regions showed that *A. pictum* had SRs comparable to those of UK native species (Figure 6), suggesting the highest potential for adapting to the changing climate in the UK. However, considering that *A. pictum* is distributed across the widest geographic range, its apparent adaptability may be linked to underlying genetic diversity that is masked in the data aggregation process: when individual species' data are highly aggregated—in this case, subspecies are aggregated to species level—key information can be lost in the averaging (Clark et al. 2011). *Acer mono* Maxim. is the major subspecies of *A. pictum* cultivated in the selected botanic gardens in this study. A recent study by Mori et al. (2024) found that this subspecies is predominantly distributed in drier sites of Northern regions in Japan and exhibits greater drought tolerance. This distinguishes it from other Japanese subspecies found in wetter regions extending to Southwestern Japan. The pattern is also observed in *A. pictum* populations in China and Korea, which largely consist of two genetically divergent and isolated groups: one distributed in cool temperate regions and the other in warm temperate regions (Guo et al. 2014; Ye et al. 2015). Such divergent speciation is most often allopatric, caused by geographic isolation (Warren et al. 2014) and can result in contrasting traits and habitat preferences. Without properly accounting for this genetic difference among populations, the estimated HS inferred from the broad community‐level summaries might not accurately reflect individual tolerance (Clark et al. 2011; Vilà‐Cabrera et al. 2019).

Pseudo‐absence sampling strategy, which is intricately linked to local geological conditions, is also an important consideration that determines the model performance and discrepancies between HS‐SR. The result that the ‘*G.E*.’ approach performed better than the ‘*E.S*.’ suggests that physical landscape elements—such as topographic variations and structural complexity—may play a more crucial role in defining species distributions. For example, even when environmental conditions are suitable for a species, certain physical landscape features can prevent species from occupation. Additionally, sampling bias might have influenced the ‘*E.S*.’ approach, particularly in areas where occurrence data collection was incomplete due to inaccessibility. Further, species‐specific characteristics may also have contributed, as in the case of *A. palmatum* , which shows relatively more distinct geographic boundaries to its distribution (Chang 1990) and occurs in environmentally uniform habitats—specifically areas with a relatively narrower range of soil properties and more homogeneous forest structure (indicated by lower EVI*dis*). In this case, *‘G.E.’* proved to be an effective pseudo‐absence sampling strategy that worked for both presence–absence predictions and HS‐SR alignment.

Lastly, geographic conditions appear to influence how variable selection affects model performance. For instance, additions of soil and land cover variables did not improve predictions for *A. palmatum*, whereas these selections improved predictions for the other two species. This pattern likely emerged due to differences in spatial scale and magnitude of variations between the studied regions: the size of study regions can constrain environmental variation and lead to more homogeneous conditions. *A. palmatum* occupied a relatively narrower range of soil yet occurred in most of the available soil types in the studied area. This contrasts with *A. davidii*, which is distributed across a wider range of soil yet occupies only part of the available soil spectrum within China. For similar reasons, ‘bio C' variables that predicted best for *A. palmatum* in its native region did not perform the same in the UK: the extreme winter temperatures that constrained distribution in its native region rarely occurred in the UK.

**FIGURE 7:**
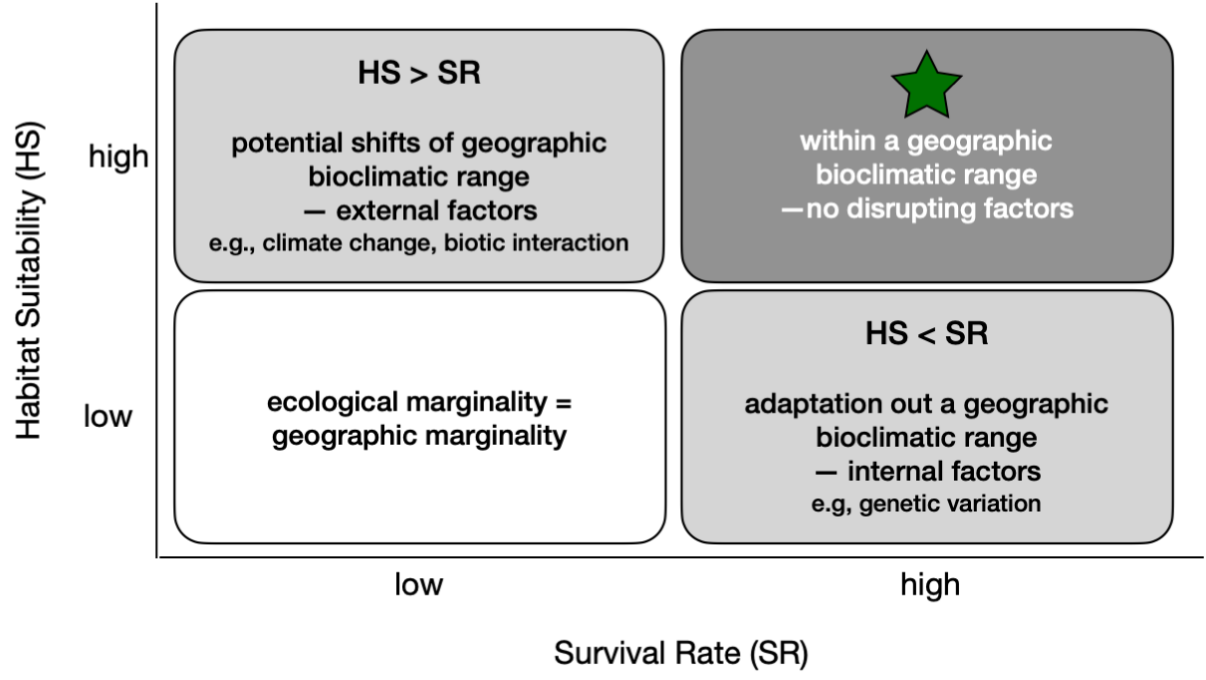
The conceptual relationship between habitat suitability (HS) and survival rate (SR).

When transferring a well‐trained model to a novel environment for predictions, some loss in predictive capacity is inevitable as the similarity to this original context decreases. Further, the HS estimations may show some discrepancies with actual individual tolerance. In essence, it is because **geographic marginality** primarily defines species distribution models whereas the true **ecological marginality**, which is more often driven by local‐scale mechanisms, tends to be lost in aggregation (Vilà‐Cabrera et al. 2019). Despite these limitations, the species distribution models at a continental scale remain valuable tools that can enhance insights when complemented by additional local information, as illustrated in Figure 7. The SRs, representing **ecological marginality** at the local scale, can inform the state of current individual tolerance. For example, HS‐SR alignment in the novel environment indicates that the species' newly occupied site is compatible with the species' geographic bioclimatic range, that is, native range. Conversely, if models overestimate HS in relation to observed SR, it suggests that there are likely external factors triggering local vulnerability, such as changes in climate patterns, increased pathogens, or abrupt land use changes. Therefore, the species has likely shifted from a position once compatible with its native range. However, if models underestimate HS in relation to observed SR, it indicates that the current location falls outside the species' geographic bioclimatic range. The persistence of the species, despite unsuitable conditions, may be attributed to underlying internal traits such as genetic variations or other ecological traits. This illustration, supported by our findings, demonstrates how species distribution models trained on native range may provide a preliminary assessment when integrated with local SR data.

### 4.3 Implications and Future Research Directions

The SRs in this study do not strictly represent demographic performance as understood in conventional population ecology. Instead, our approach is more similar to a common garden experiment assessing specific‐period climate tolerance at the individual level, as we used translocated individuals with no tracked records of reproductive capacity. In conventional ecology, the individual‐level responses have been less of interest. However, the SRs of ex‐situ species in this study provide a new context that extends beyond the species' geographic bioclimatic range, serving as a way to test **ecological marginality** (Takola and Schielzeth 2022; Vetaas 2002). Although these SRs capture only one aspect of **ecological marginality**—climate tolerance during the specific period, they complement coarse‐scaled modelling by revealing gaps that suggest the presence of hidden influencing factors, often missed in the data aggregation process.

Moreover, despite its limited sample size, the SRs conditioned on phylogeny demonstrated that member species within sect. *Macrantha*, where *A. davidii* belongs, significantly differed from the rest of the species (Table 1). This is a particularly interesting case as it suggests the presence of the **phylogenetic signal** (Pearman et al. 2008). Linking low SRs of *A. davidii* to phylogeny leads to new questions for future studies. If member species within the section exhibit similar climate tolerance despite non‐overlapping distributions in environmental space, their apparent niche differences may not be a true representation. It may be necessary to trace biogeographic history where geographic isolation has led to speciation (Warren et al. 2014). This is a possible scenario as *A. davidii* is more closely related to North American species. Peterson (2011) also pointed out how non‐overlapping environments can lead to false conclusions about niche differences if species' accessibility to other environments is not considered. The temporal scale at which niches are defined and compared is therefore a critical consideration. These evolutionary patterns also prompt an exploration into common phenotypic or ecotypic traits that might contribute to similar survivorship, beyond just environment‐centric approach such as niche similarities. Such hidden traits may not be easily detectable unless these conditions are tested at individual level on the common environment, as also highlighted by Clark et al. (2011). Additionally, a test that matches species' different phenotypes to environmental gradients could further advance this research direction, as illustrated by Trappes et al. (2022).

## 5 Conclusion

Most predictive species distribution models rely on correlative relationships between environmental occurrences. However, correlative models have intrinsic limitations, especially for extrapolated projections, due to two key discrepancies: (1) between true population density and sampling density, and (2) between observed biogeographic range and ecological range, influenced by various non‐climatic factors such as biotic interactions and inaccessibility. Our findings highlight the significance of incorporating local‐scale individual survival response data to assess modelled predictions in revealing these discrepancies. These survival responses were not random but rather exhibited patterns within a phylogenetic framework. We observed similar climate tolerance among phylogenetically closer species, although their biogeographical distributions are not necessarily overlapping. In addition, the discrepancies between predictions (HS) and observed SRs provide clues to uncover additional key factors that are often overlooked in the conventional macroecological modelling approach, such as genetic variation, local adaptations, and short‐term climate stochasticity. Distinguishing these independent factors that condition individual responses, in conjunction with macro biogeographic distributions, may improve our predictive modelling in future studies. This approach, in particular, should incorporate species‐specific traits that are conserved across phylogenetic lineages as these traits govern species' responses to environmental change. Understanding how these intrinsic traits interact with external driving forces to shape ecological communities has long been a central question in ecology. However, balancing these two aspects has also been hindered by practical logistical issues, such as difficulties in data collection and the development of a shared knowledge platform on a global scale. In this context, we present the potential of leveraging living collections data from the botanic gardens. These collections, sourced from diverse provenance, can serve as an excellent testing platform for comparative experiments across botanic gardens. Further, each botanic garden, as a distinct reference point within the biogeographic region, can effectively manage trait data requiring long‐term efforts. Such platforms can have impact when acting collectively, transforming their roles to extend from local conservation to serving a broader global community in facing climate change.

## Author Contributions

**Eun Hye Kim:** conceptualization (lead), data curation (lead), formal analysis (lead), methodology (equal), validation (equal), visualization (lead), writing – original draft (lead). **James D. Hitchmough:** conceptualization (supporting), supervision (lead), writing – review and editing (supporting). **Ross W. Cameron:** conceptualization (supporting), resources (supporting), supervision (lead). **Volker Bahn:** methodology (equal), validation (supporting), writing – review and editing (supporting). **Franziska Schrodt:** methodology (equal), validation (supporting), writing – review and editing (supporting).

## Conflicts of Interest

The authors declare no conflicts of interest.

## Supporting information


Appendix S1.

Appendix S2.

Appendix S3.

## Data Availability

The data that supports the findings of this study are available in the Supporting Information of this article. These data were derived from the following resources available in the public domain: gbif (https://www.gbif.org), CHELSA (https://chelsa‐climate.org), ISRIC Soil Data Hub (https://data.isric.org), EarthEnv (https://www.earthenv.org) .
