# Supplementary material for: Relating Habitat Suitability and Survival Rates in a Phylogenetic Framework
Source: Ecol Evol. 2025 Mar 2;15(3):e71030. doi: 10.1002/ece3.71030 (PMC11872369; doi:10.1002/ece3.71030)
Supplement: Supplementary file 2 — Appendix S2. [file ECE3-15-e71030-s002.docx]

**Supplementary figures and tables**

**Table 1**. List of all variables used for bio selection A, B, and C

| Category | Variable | Description | *A. davidii* | *A. palmatum* | *A.pictum* |
| --- | --- | --- | --- | --- | --- |
| Climate | bio2 | Mean diurnal air temperature | A, B, C | A, C | A, B, C |
|  | bio3 | Isothermality | A, B, C | B, C | A, B, C |
|  | bio4 | Temperature seasonality | A, B | A | A, B |
|  | bio5 | Mean daily maximum air temperature of the warmest month |  | A | A |
|  | bio6 | Mean daily minimum air temperature of the coldest month | C | C | C |
|  | bio8 | Mean daily mean air temperature of the wettest quarter |  | A, B | B |
|  | bio9 | Mean daily mean air temperature of the driest quarter | C | A, C | C |
|  | bio10 | Mean daily mean temperature of the warmest quarter | A, B |  |  |
|  | bio15 | Precipitation seasonality | A, B | A, B |  |
|  | bio16 | Mean monthly precipitation of the wettest quarter |  | A | A |
|  | bio18 | Mean monthly precipitation of the warmest quarter | A |  |  |
|  | bio19 | Mean monthly precipitation of the coldest quarter |  | A, B | A, B |
|  | CMIgs | Mean monthly climate moisture index during the growing season | B | B | B |
|  | CMIspr | Mean monthly CMI from March to May | C | C | C |
|  | CMIsmr | Mean monthly CMI from June to August | C | C | C |
|  | PETgs | Mean monthly potential evapotranspiration during the growing season | B | B | B |
|  | PETspr | Mean monthly PET from March to May | C | C | C |
|  | PETsmr | Mean monthly PET from June to August | C | C | C |
|  | rsds | Mean monthly surface downwelling shortwave flux in air | B, C | B, C | B, C |
|  | ngd | Number of growing degree days at which mean air temperature > 5°C | B, C |  | B |
| Soil | Silt | Silt contents | O | O | O |
|  | Clay | Clay contents | O | O | O |
|  | Bulk density | Bulk density of the fine earth fraction | O | O | O |
|  | pH | Soil pH | O | O | O |
| Land cover | EVI*max*, | Mean monthly maximum EVI of the year | O | O | O |
|  | EVI*dis* | Difference in EVI between adjacent pixels | O | O | O |


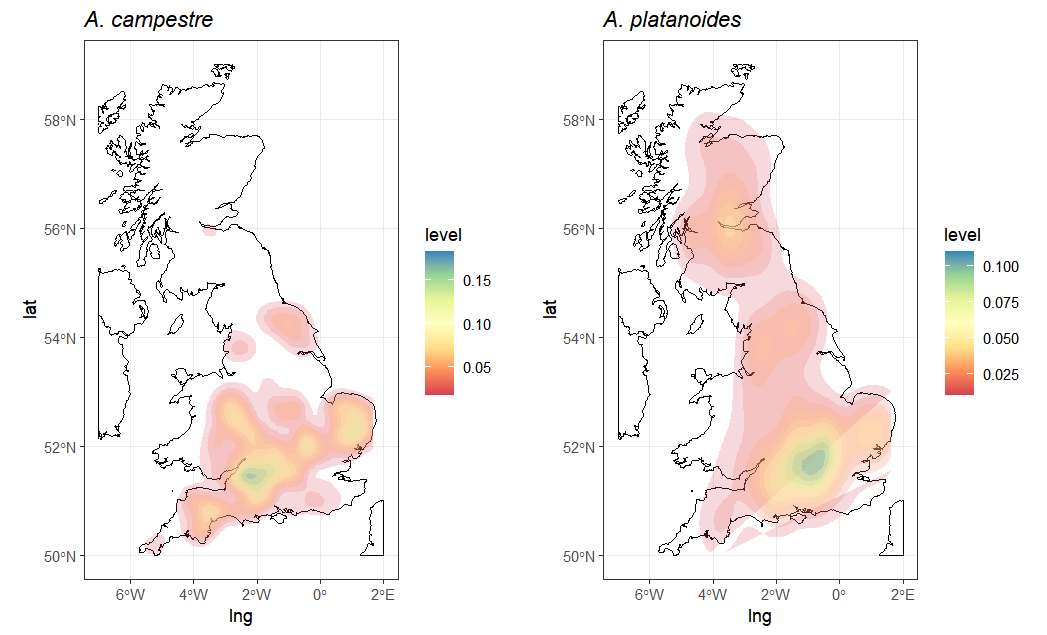


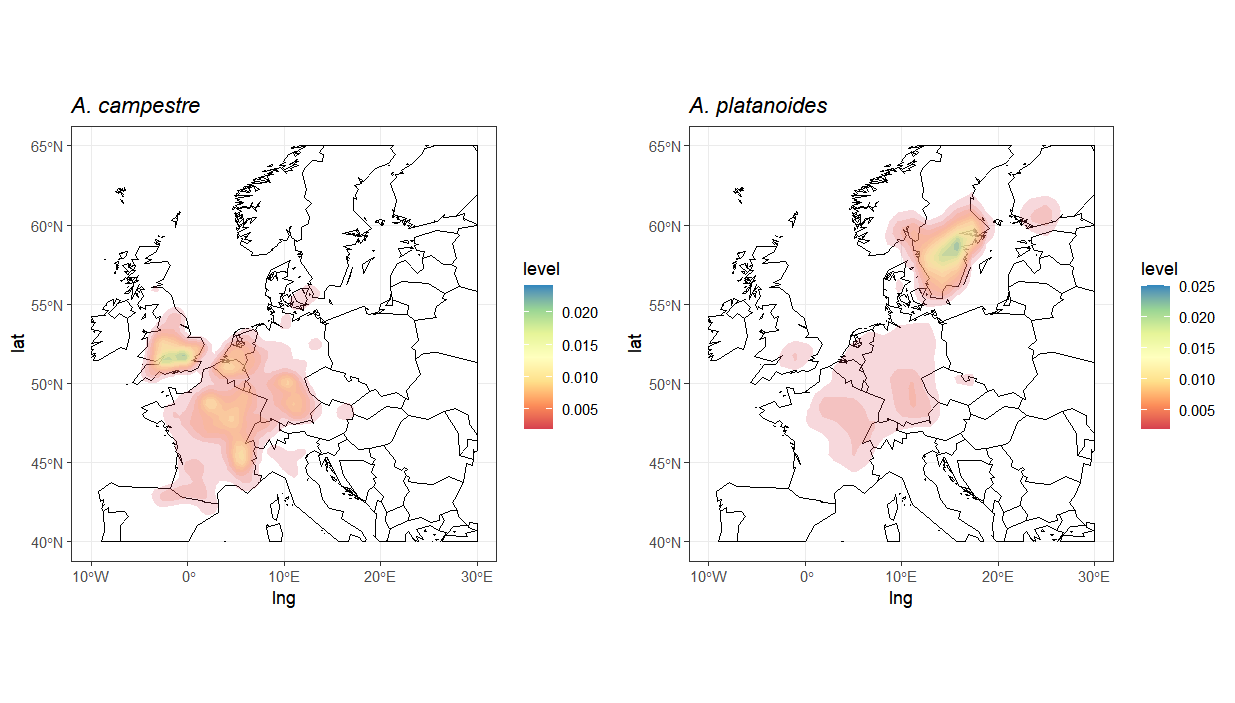
**Figure 1**. Occurrence density plots of *A. campestre* (n=14,508) and *A. platanoides* (n=3,036) in the UK (top) and *A. campestre* (*n*=66,712) and *A. platanoides* (*n*=66,482) in the Europe (bottom).

**Table 2.** Test results of niche similarity with *A. campestre*. Metrics are indicated by D, Schoener’s D (Schoener, 1970) and by I, Warren’s I (Warren et al., 2008). Bold numbers indicate significant niche overlap between species for the selected environmental variables (values more similar than expected by random chance) with statistical significance as in **, P < 0.01, ∗, *P* ＜ 0.05, ‘, *P* ＜ 0.1

| Vars. | *A. davidii - A. campestre* | | | | *A. palmatum - A. campestre* | | | | *A. pictum - A. campestre* | | | |
| --- | --- | --- | --- | --- | --- | --- | --- | --- | --- | --- | --- | --- |
|  | UK | | Europe | | UK | | Europe | | UK | | Europe | |
|  | D | I | D | I | D | I | D | I | D | I | D | I |
| bio A | **0.06*** | **0.20*** | 0.22 | 0.44’ | 0.00 | 0.00 | 0.18 | 0.36 | 0.02 | 0.05 | 0.08 | 0.16 |
| bio B | 0.06 | 0.19 | 0.25 | 0.49 | 0.00 | 0.00 | 0.18 | 0.35 | 0.14 | 0.32 | 0.12 | 0.31 |
| bio C | **0.16*** | **0.35*** | 0.27 | 0.53 | 0.00 | 0.00 | 0.09 | 0.24 | 0.12’ | 0.30’ | 0.13 | 0.36 |
| Soil | 0.25 | 0.40 | 0.32’ | 0.54’ | 0.24 | 0.43 | 0.21 | 0.40 | 0.17 | 0.32 | 0.17 | 0.34 |
| EVI | 0.49 | 0.74 | 0.49 | 0.76 | **0.52*** | **0.75*** | 0.37 | 0.62 | 0.56’ | 0.80’ | 0.59’ | 0.82’ |

**Table 3.** Test results of niche similarity with *A. platanoides*. Metrics are indicated by D, Schoener’s D (Schoener, 1970) and by I, Warren’s I (Warren et al., 2008). Bold numbers indicate significant niche overlap between species for the selected environmental variables (values more similar than expected by random chance) with statistical significance as in**, P < 0.01, ∗, *P* ＜ 0.05, ‘, *P* ＜ 0.1

| Vars. | *A. davidii – A. platanoides* | | | | *A. palmatum – A. platanoides* | | | | *A. pictum – A. platanoides* | | | |
| --- | --- | --- | --- | --- | --- | --- | --- | --- | --- | --- | --- | --- |
|  | UK | | Europe | | UK | | Europe | | UK | | Europe | |
|  | D | I | D | I | D | I | D | I | D | I | D | I |
| bio A | 0.07’ | **0.21*** | 0.08 | 0.19 | 0.00 | 0.00 | 0.34 | 0.60 | 0.01 | 0.04 | 0.24’ | 0.50’ |
| bio B | 0.06 | 0.18 | 0.23’ | 0.48’ | 0.00 | 0.00 | 0.45’ | **0.71*** | 0.19 | 0.36 | 0.36’ | **0.67*** |
| bio C | **0.20*** | **0.41*** | 0.23 | 0.46 | 0.00 | 0.00 | 0.28’ | 0.54’ | 0.16 | 0.37 | **0.39*** | **0.68*** |
| Soil | 0.07 | 0.19 | **0.07*** | **0.25**** | 0.23 | 0.40 | 0.08 | 0.24 | 0.10 | 0.20 | 0.14 | 0.33 |
| EVI | 0.48 | 0.71 | 0.47 | 0.75 | 0.54’ | 0.76 | 0.38 | 0.62 | **0.62**** | **0.79*** | **0.54*** | 0.78’ |
